# Assessing the optimal MRI descriptors to diagnose Ménière’s disease and the added value of analysing the vestibular aqueduct

**DOI:** 10.1007/s00330-024-10587-w

**Published:** 2024-02-07

**Authors:** Steve Connor, Irumee Pai, Philip Touska, Sarah McElroy, Sebastien Ourselin, Joseph V. Hajnal

**Affiliations:** 1 School of Biomedical Engineering and Imaging Sciences, King’s College London, St Thomas’ Hospital, London, SE1 7EH UK; 2 Department of Neuroradiology, King’s College Hospital, London, SE5 9RS UK; 3 Department of Radiology, Guy’s Hospital and St Thomas’ Hospital, London, SE1 9RT UK; 4 Department of Ear, Nose and Throat Surgery, Guy’s and St Thomas’ Hospital, London, SE1 9RT UK; 5 MR Research Collaborations, Siemens Healthcare Limited, Camberley, UK

**Keywords:** Magnetic resonance imaging, Endolymphatic hydrops, Inner ear, Vestibular aqueduct, Odds ratio

## Abstract

**Objectives:**

To evaluate the diagnostic performance and reliability of MRI descriptors used for the detection of Ménière’s disease (MD) on delayed post-gadolinium MRI. To determine which combination of descriptors should be optimally applied and whether analysis of the vestibular aqueduct (VA) contributes to the diagnosis.

**Materials and methods:**

This retrospective single centre case-control study evaluated delayed post-gadolinium MRI of patients with Ménièriform symptoms examined consecutively between Dec 2017 and March 2023. Two observers evaluated 17 MRI descriptors of MD and quantified perilymphatic enhancement (PLE) in the cochlea. Definite MD ears according to the 2015 Barany Society criteria were compared to control ears. Cohen’s kappa and diagnostic odds ratio (DORs) were calculated for each descriptor. Forward stepwise logistic regression determined which combination of MRI descriptors would best predict MD ears, and the area under the receiver operating characteristic curve for this model was measured.

**Results:**

A total of 227 patients (mean age 48.3 ± 14.6, 99 men) with 96 definite MD and 78 control ears were evaluated. The presence of saccular abnormality (absent, as large as or confluent with the utricle) performed best with a DOR of 292.6 (95% confidence interval (CI), 38.305–2235.058). All VA descriptors demonstrated excellent reliability and with DORs of 7.761 (95% CI, 3.517–17.125) to 18.1 (95% CI, 8.445–39.170). Combining these saccular abnormalities with asymmetric cochlear PLE and an incompletely visualised VA correctly classified 90.2% of cases (sensitivity 84.4%, specificity 97.4%, AUC 0.938).

**Conclusion:**

Either absent, enlarged or confluent saccules are the best predictors of MD. Incomplete visualisation of the VA adds value to the diagnosis.

**Clinical relevance statement:**

A number of different MRI descriptors have been proposed for the diagnosis of Ménière’s disease, but by establishing the optimally performing MRI features and highlighting new useful descriptors, there is an opportunity to improve the diagnostic performance of Ménière’s disease imaging.

**Key Points:**

• A comprehensive range of existing and novel vestibular aqueduct delayed post-gadolinium MRI descriptors were compared for their diagnostic performance in Ménière’s disease.

• Saccular abnormality (absent, confluent with or larger than the utricle) is a reliable descriptor and is the optimal individual MRI predictor of Ménière’s disease.

• The presence of this saccule descriptor or asymmetric perilymphatic enhancement and incomplete vestibular aqueduct visualisation will optimise the MRI diagnosis of Ménière’s disease.

**Supplementary Information:**

The online version contains supplementary material available at 10.1007/s00330-024-10587-w.

## Introduction

Ménière’s disease (MD) is an inner ear condition characterised by the clinical presentation of low- to mid-frequency hearing loss, episodic vertigo and fluctuating aural symptoms. The increased worldwide application of delayed post-gadolinium magnetic resonance imaging (MRI) for the diagnosis of MD has resulted in a shift in the diagnostic paradigm. The detection of MD-affected ears is based on the presence of various qualitative or semi-quantitative MRI descriptors and grading scales which define expansion of the endolymphatic space (ES), termed endolymphatic hydrops (EH). However, there is no universal consensus on which specific MRI features best distinguish affected ears [1].

Previous studies have generally applied small numbers of MRI descriptors within individual grading systems [2–6], with only a few authors comparing the diagnostic performance of different grading systems in the same patient cohort [2, 7–10]. Thus, the evaluation of differences in diagnostic performance across the range of MRI descriptors has involved pooled comparisons between heterogenous studies, and this approach is subject to bias [1]. This matter may be further explored by directly comparing the diagnostic performance of a comprehensive range of different MRI descriptors within a single large cohort. Furthermore, there remains uncertainty as to how MRI descriptors may be optimally combined in order to corroborate the diagnosis of MD and only limited combinations have been evaluated. Whilst the presence of both vestibular EH and increased perilymphatic enhancement (PLE) has demonstrated improved diagnostic performance [6, 11], further combinations of MR descriptor variables remain untested [1].

There is also the potential for new MRI descriptors to enhance the prediction of MD provided by existing imaging features [1]. Whilst it has been postulated that the vestibular aqueduct (VA) is integral to the pathophysiology of MD [12–15] and imaging abnormalities have been described in MD [16–18], there is limited data on its post-gadolinium MRI appearances [19–23]. Variation in the visibility and signal of the VA [18, 21–23] may act as potential biomarkers for MD ears, and their added value should be assessed.

This study aimed to explore and quantify the reliability, performance and optimal combination of MRI descriptors, in order to detect MD ears on delayed post-gadolinium MRI. In addition, it intended to evaluate the diagnostic accuracy of novel MRI features of VA and their potential contribution to the diagnosis of MD.

## Methods

### Patients

**Fig. 1:**
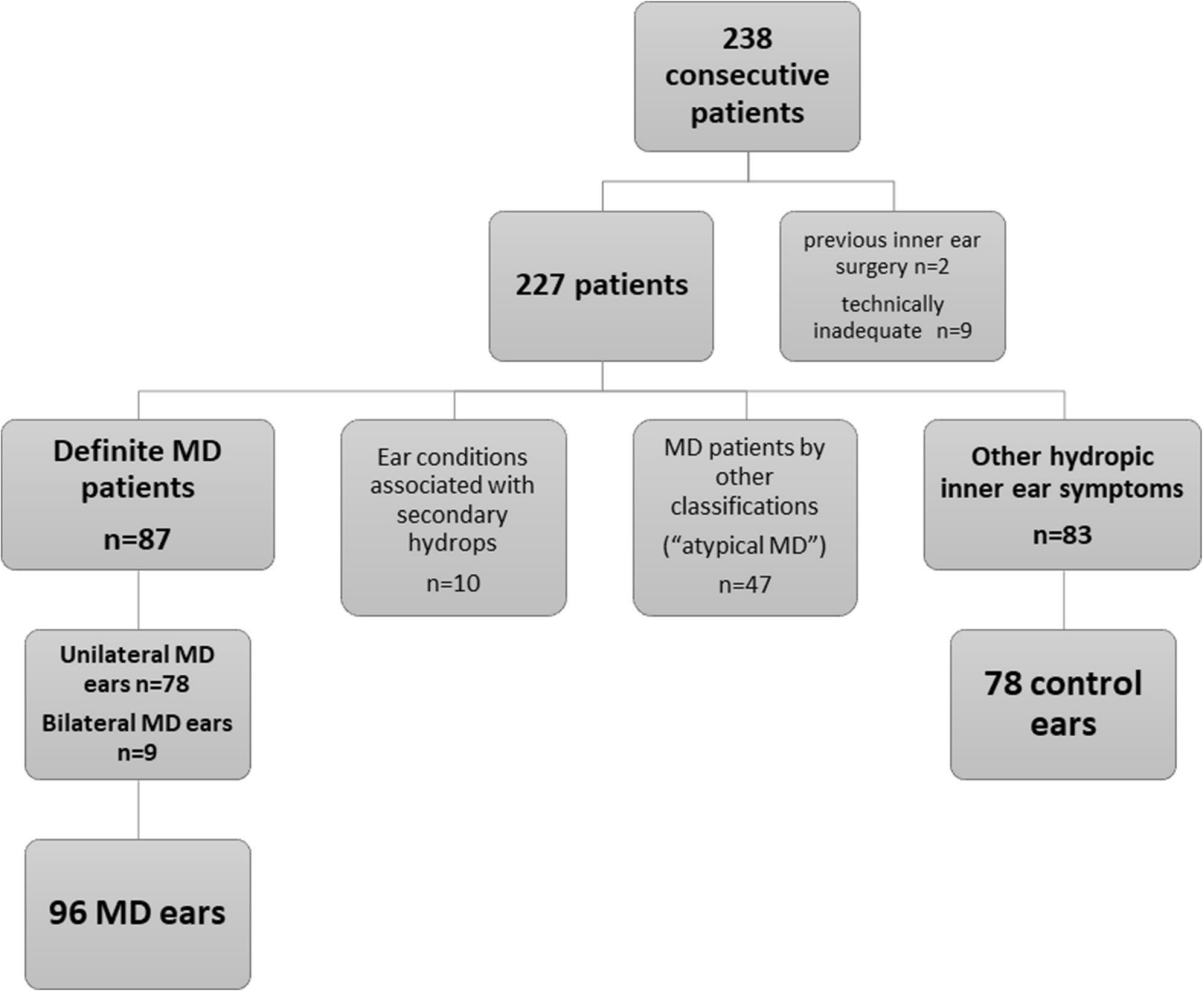
Flowchart demonstrating the selection process for the 96 Ménière’s disease and 78 control ears

The study was approved by the institutional ethical committee (GSTT Electronic Record Research Interface, IRAS ID: 257283, Rec Reference: 20/EM/0112). This retrospective case-controlled cross-sectional study evaluated consecutively examined patients who underwent delayed post-gadolinium MRI for the evaluation of endolymphatic hydrops between December 2017 and March 2023. Patients presented with symptoms of inner ear hydrops such as episodic vertigo, sudden-onset or fluctuating sensorineural hearing loss (SNHL), aural fullness and tinnitus. Patient details were retrieved using the PACS system search function (Sectra AB). A priori exclusion criteria were previous inner ear operations, inadequate contemporary clinical details for diagnostic classification and technically inadequate MR imaging (Table 1; Fig. 1).

**Table 1:** Summary of the demographics of the study sample

227 patients	
Sex	128 women/99 men
Age	Mean 48.3 ± 14.6 Median 48 years (range 13–88 years)
Duration of symptoms	Mean 88.3± 101 Median 43 months (range 1–456 months)
MD diagnosis	87 definite MD patients 47 patients with atypical MD

### Clinical data and classification

Two observers (S.C., I.P.) reviewed the contemporary clinical and audiometric data by consensus, blinded to imaging findings. Clinical review was required to be within 6 months and audiometry within 12 months prior to the MRI study. Definite MD was classified according to the current 2015 Barany Society criteria (Supplementary Table 1) [24]. Definite MD was not diagnosed if there was a clinical diagnosis of secondary hydrops. Control ears were obtained from patients without any criteria for either MD or “atypical MD” [25–30] and who had (a) no Ménière’s-type vertigo, (b) normal hearing (thresholds ≤ 20 dBHL (decibels hearing loss) at 0.5, 1, 2 and 4 kHz) or (c) isolated high-frequency sensorineural hearing loss ≥20 dBHL at ≥ 6 kHz (Supplementary Table 1). Intratympanic therapy (ITT) with gentamicin or corticosteroid prior to the MRI study was recorded.

### MRI protocol and technique

MRI for inner ear EH was performed on a 3-Tesla Siemens Magnetom® Skyra scanner with a 64-channel head coil. MRI was performed 4 h after the intravenous administration of gadoterate meglumine (0.2 mmol/kg) with an isotropic (0.7mm voxel) high-resolution three-dimensional inversion recovery (IR) sequence with real reconstruction. An additional T2-weighted sampling perfection with application-optimised contrasts using different flip angle evolution (SPACE) sequences was performed. Siemens product sequences were used with parameters as set out in Table 2.

**Table 2:** MRI parameters for the 3D-real IR and T2-SPACE sequence

	3D-real IR	T2-SPACE
Repetition time	6000 ms	1000 ms
Echo time	180 ms	125 ms
Inversion time	2000 ms	NA
Number of excitations	1	2
Refocusing flip angle	180° (constant)	100°
Echo train length	27	52
Pixel spacing	0.7 mm	0.31 mm
Slice thickness	0.7 mm	0.3 mm
Matrix size	256 × 240	262 × 512
Field of view	178 mm	80 × 160 mm

### Extraction and definition of qualitative MRI descriptors

**Fig. 2:**
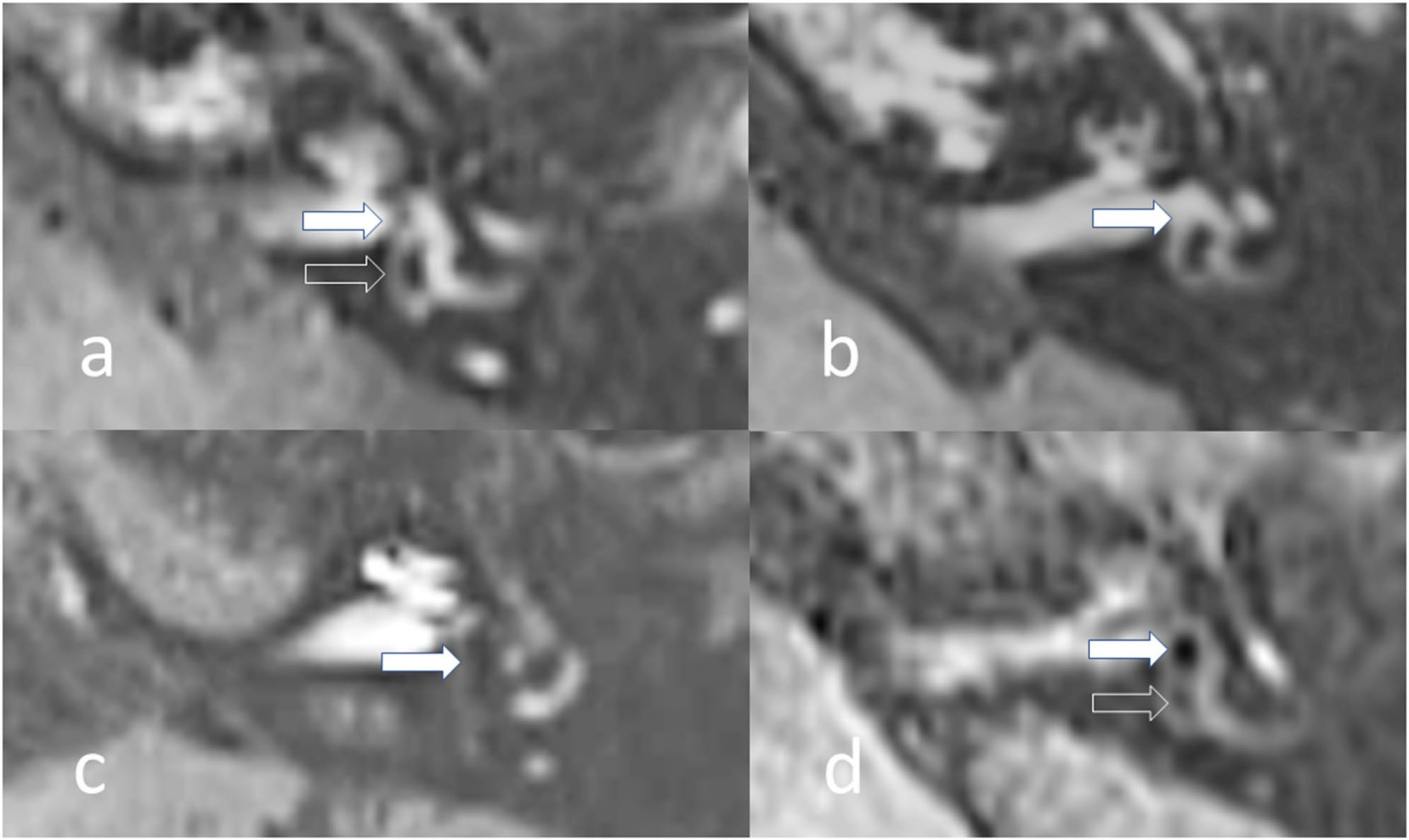
Delayed post-gadolinium 3D-real IR axial images angled through the inferior vestibule demonstrating saccule-related MRI descriptors. **a** Normal appearances with the smaller saccule (filled arrow) anterior to the utricle (open arrow). **b** Absent saccule with the expected location indicated (filled arrow). **c** Saccule confluent with the utricle (filled arrow). **d** Saccule (filled arrow) larger than the utricle (open arrow)

**Fig. 3:**
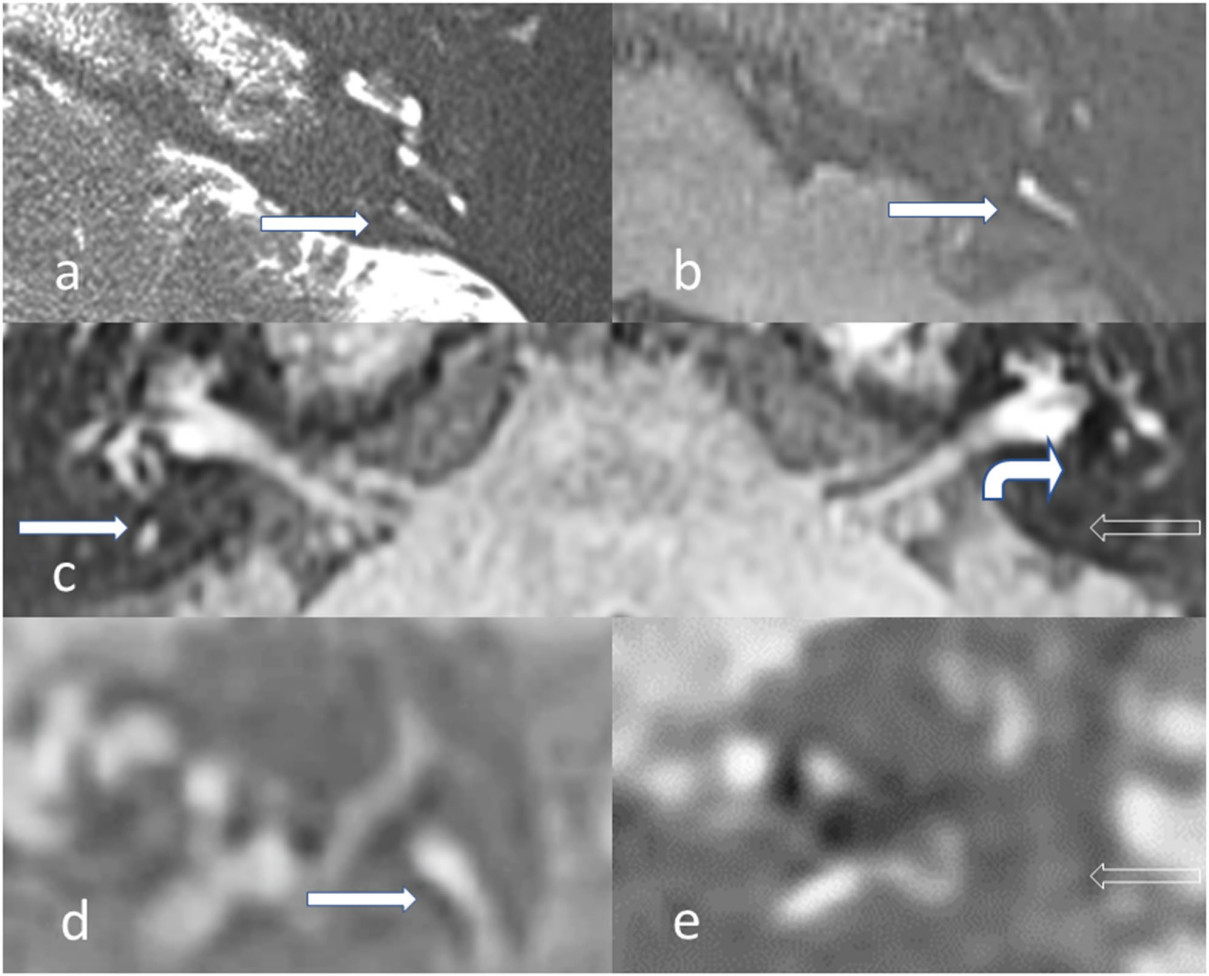
Vestibular aqueduct appearances on axial and sagittal images. **a** T2w SPACE and (**b**) delayed post-gadolinium 3D-real IR axial images showing a “fan shaped” vestibular aqueduct which is well demonstrated on a single axial section (filled arrows). **c** Delayed post-gadolinium 3D-real IR axial image in a patient with left-sided unilateral MD. The asymptomatic right ear shows a short segment of the “tubular” vestibular aqueduct (filled arrow). The symptomatic left ear demonstrates vestibulo-cochlear endolymphatic hydrops and replacement of the vestibular perilymph (curved filled arrow), with no vestibular aqueduct visualised (open arrow). Delayed post-gadolinium 3D-real IR sagittal images of the (**d**) right ear and (**e**) left ear in the same patient. The “tubular” vestibular aqueduct is better depicted on the right due to its angulation (filled arrow in **d**) whilst the left vestibular aqueduct remains non-visualised (open arrow in **e**)

**Fig. 4:**
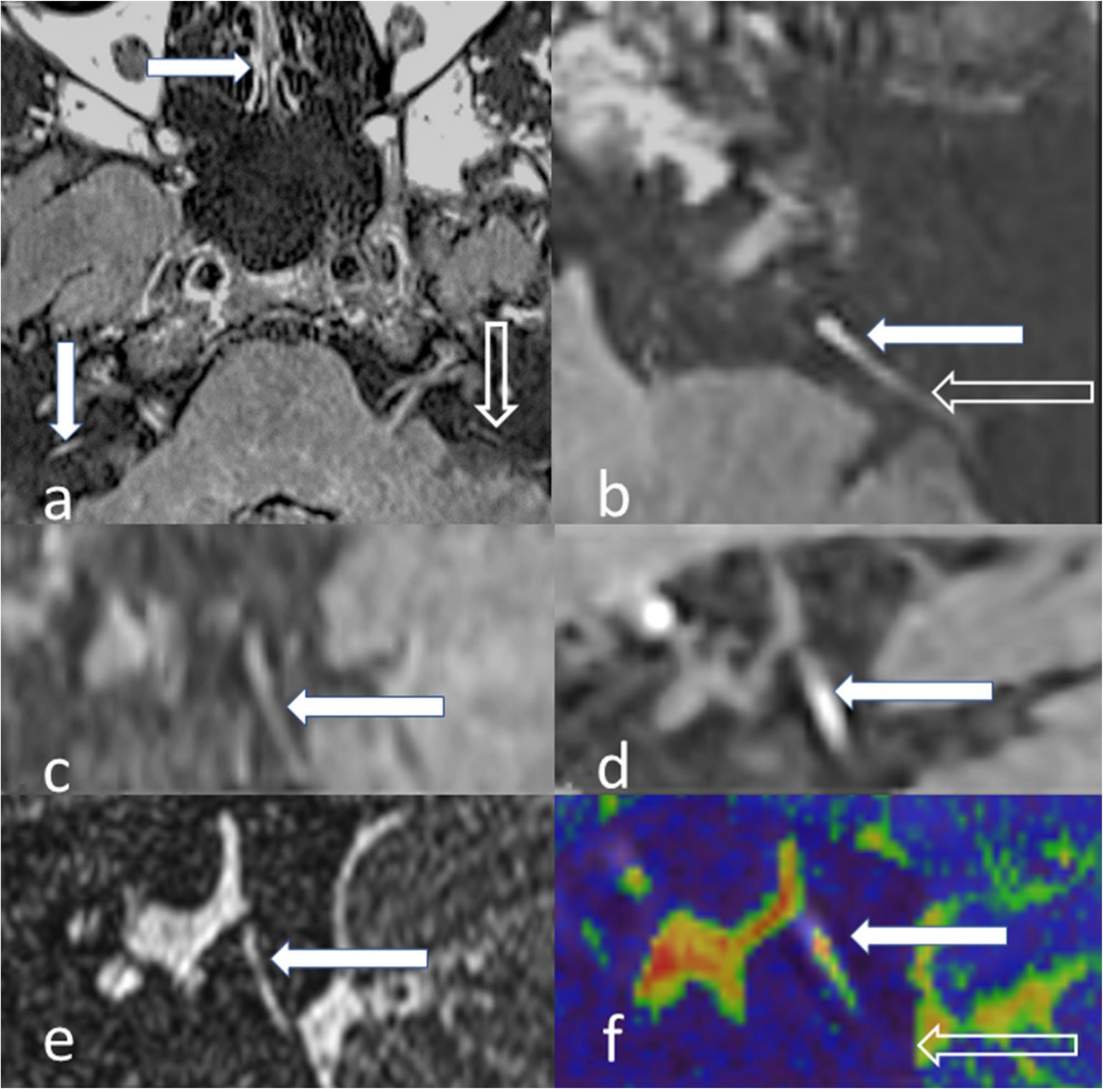
Vestibular descriptor analysis. **a** Delayed post-gadolinium 3D-real IR axial image demonstrates a right vestibular aqueduct (filled vertical arrow) to be as hyperintense as the enhancing nasal mucosa (horizontal filled arrow) whilst the left vestibular aqueduct (open arrow) is not hyperintense. **b** Delayed post-gadolinium 3D-real IR axial image shows an axial image with a completely visualised vestibular aqueduct. Note: The medial component (filled arrow) indicates it to be classified as hyperintense, despite the intermediate signal lateral portion (open arrow). **c** Delayed post-gadolinium 3D-real IR sagittal image illustrates a completely visualised vestibular aqueduct (filled arrow) which is not hyperintense. **d** Delayed post-gadolinium 3D-real IR sagittal image shows a hyperintense vestibular aqueduct; however, its relationship to the posterior petrous bone is better demonstrated in sagittal (**e**) T2w SPACE and (**f**) fused 3D-real IR and T2w SPACE images, which allow it to be more confidently classified as incompletely visualised (open arrow indicating posterior petrous bone)

The MRI grading scales or unique individual EH descriptors which had been previously applied to ≥ 3 published diagnostic accuracy studies of MD were collated [1]. This yielded four grading scales (Nakashima, Barath, Bernaerts and Kahn) (Supplementary Table 2), and one additional descriptor of asymmetric PLE [1, 3–6]. The MRI descriptors extracted were classified according to whether they evaluated the cochlea, superior vestibular endolymphatic space (VES) or inferior VES (Table 3). Single cochlear grades were applied since they only slightly differ between grading systems. Since “saccule as large as the utricle” cannot be accurately evaluated when “confluent with the utricle”, these were combined as “saccule as large as or confluent with the utricle” to avoid missing values. An exploratory saccular descriptor was added as “saccule absent, as large as or confluent with the utricle” (Fig. 2) [2]. MRI descriptors pertaining to the decreased conspicuity of the VA were newly defined as “non-visualised VA”, “incompletely visualised VA” or “no hyperintensity VA” (Table 3; Figs. 3 and 4).

**Table 3:** Description of MRI descriptors and associated grading systems

Descriptor	Corresponding grading scale		Details of analysis
Cochlear			
Grade 1 (or above)	Barath [3]/Bernaerts [4]	Nodular cochlear duct enlargement with cochlear duct sparing part of scala vestibuli	Axial through mid-modiolar level
Grade 2		Linear cochlear duct enlargement with cochlear duct replacing scala vestibuli	
Superior vestibule			
> 33% VES area relative to TV area (superior)	Nakashima [6]	There is > 33% area of the combined VES relative to the TV fluid area on an axial cross-section	Axial parallel to and at the inferior aspect of lateral semi-circular canal where it is visualised more than 240°
> 50% VES area relative to TV area (superior)	Nakashima [6]	There is > 50% area of the combined VES relative to TV fluid area	
No lateral SCC ampullary PS visible	Kahn [5]	The extension of the VES into the ampulla of the lateral SCC is dilated so no PS is visible	Axial parallel to and at the level of the lateral semi-circular canal
Lateral SCC posterior limb VES extension	Kahn [5]	There is protrusion of the VES into the non-ampullated posterior limb of the lateral SCC	
PS not visible (superior)	Kahn [5]	The VES replaces the TVS and no PS is visible	
Inferior vestibule			
Saccule as large as the utricle	Bernaerts [4]/Kahn [5] as described by Attye [2]	Termed “SURI”. The ratio of the area of the saccule to the area of the utricle is ≥ 1	Axial through the widest part of the vestibule and oblique sagittal in line of the vestibule
Saccule confluent with the utricle	Bernaerts [4]	There is confluence of the saccule and utricle (with no acute angle at its interface)	Axial angled from posterior limb of lateral semi-circular to modiolus through the widest part of the inferior vestibule
> 50% VES area relative to TV area (inferior)	Barath [3]	There is >50% area of the combined VES relative to TV fluid area	
PS not visible (inferior)	Bernaerts [4]	The VES replaces the TV fluid area and no PS is visible	
VES contacting the oval window	Kahn [5] as described by Conte et al [31]	Termed “VESCO”. The VES contacts the oval window and effaces the adjacent perilymphatic space	Axial parallel to lateral SCC and oblique sagittal planes (parallel to superior SCC)
Vestibular aqueduct			
Non-visualised VA	NA	No structure anatomically consistent with the VA	Identified as a linear structure in either axial or sagittal planes with scrolling to visualise the whole course of the obliquely orientated VA
Incompletely visualised VA	NA	No VA seen extending uninterrupted from the level of the posterior vestibule to the posterior petrous pyramid	
No hyperintensity VA	NA	No VA which returns signal equal to or higher than the nasal mucosa	

*VESCO* vestibular endolymphatic space contacts the oval window, *SURI* saccule to utricle area ratio inversion, *VES* vestibular endolymphatic space, *TV* total vestibular, *PS* perlymphatic space, *SCC* semi-circular canal, *VA* vestibular aqueduct

### Imaging analysis

The delayed post-gadolinium 3D-real IR sequence was sequentially reformatted to the planes as defined in Table 3 and Supplementary Table 2 on a PACS workstation (Sectra Workstation, Sectra AB), with T2w SPACE images used for anatomical correlation as required.

The images were reviewed with standardised magnification and window settings. Two radiologists (P.T. and S.C.), with 6 and 24 years of subspecialty head and neck radiology experience, independently evaluated the MRI studies. The qualitative MRI descriptors were analysed according to the defined criteria (Table 3) and the four grading scales were also scored based on the observed descriptors. Imaging review was performed whilst blinded to clinical diagnosis, and the two observers achieved consensus when different scores were obtained.

**Fig. 5:**
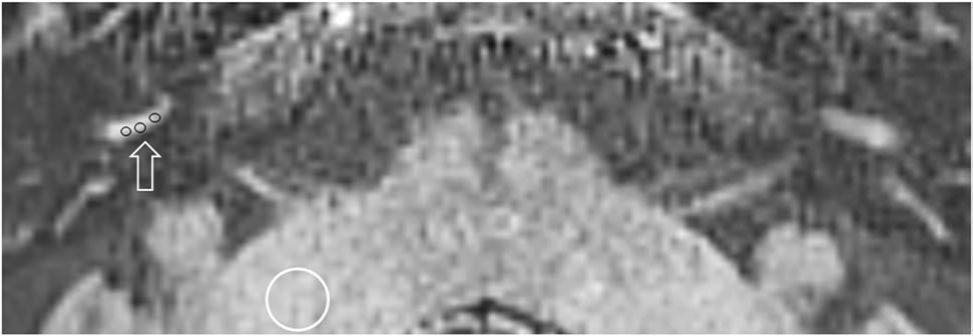
Quantitative analysis of cochlea PLE. The mean of three 1 mm^2^ regions of interest (ROI) in the scala tympani of the inferior segment of basal turn (black circles and indicated by open arrow) and the mean value was divided by a reference measurement from a 30 mm^2^ ROI in the middle cerebellar peduncle (white circle)

A quantitative analysis of PLE was measured as the signal intensity ratio of the cochlear basal turn relative to the ipsilateral middle cerebellar peduncle [11] as an internal reference (Fig. 5). A mean of the values obtained by the two observers was used for further analysis.

After review of the VA descriptors on delayed post-gadolinium 3D-real IR, the observers referred to the T2w SPACE sequence and any sub-millimetric computed tomography (CT) imaging to corroborate the anatomical location of the VA.

### Statistics

Statistical analyses were performed using SPSS® Statistics 25.0 (IBM®). Inter-rater reliability of the qualitative MRI descriptors was evaluated with Cohen’s kappa test. Since the variable was not normally distributed on the Shapiro-Wilk’s test (*p* < 0.05), the correlation between quantitative cochlear measurements for the two observers was evaluated with Spearman’s linear weights rank-order correlation. Agreement between the two observers for the four grading scales was performed with a weighted kappa (*κw)* with linear weights.

The 2 × 2 contingency tables were created for the presence of MRI descriptors in definite MD and control ears, with the chi-squared test of independence conducted (significance level of *p* < 0.00294 to account for multiple comparisons). The diagnostic odds ratios (DORs), sensitivity and specificity were calculated. A Mann-Whitney *U* test determined any difference in the quantitative PLE measurements in MD ears. The chi-squared test of independence compared the scores for the four grading scales between MD ears and controls.

A binomial forward stepwise logistic regression was performed to determine the optimal combination of MRI predictors of definite MD. Descriptors with reliability kappa score < 0.6 and variance inflation factor > 5 were excluded. According to Vittinghoff and McCulloch [32], the sample size allowed the inclusion of all the MRI-based predictors in the model. At each step, the most highly correlated remaining variable was added, and the *p*-value threshold of 0.05 was used to set a limit on the number of variables used in the final model. Area under the receiver operating characteristic (ROC) curve was calculated. The forward stepwise logistic regression was repeated after removing MD ears which had undergone ITT.

## Results

### Descriptive data of cohort

There were 238 consecutive patients referred by our tertiary ENT unit for delayed post-gadolinium MRI. Patients with previous inner ear surgery (*n* = 2) and technically inadequate MR imaging (*n* = 9) were excluded from the study (Fig. 1).

Of the remaining 227 patients (128 female, 99 males; age mean 48.3 ± 14.6 years; duration of symptoms mean 88.3 ± 101 months), there were 87 patients with definite MD. This comprised 78 patients with unilateral MD, of which five also had contralateral “atypical” MD ears, and nine patients with bilateral MD. Hence, there were a total of 96 definite MD ears, and nine of these had undergone prior ITT. There were ten patients with conditions associated with secondary hydrops and 47 patients with “atypical” MD (40 unilateral, 7 bilateral) (Supplementary Table 1) [24–30]. The remaining 83 patients presented with audio-vestibular symptoms without diagnostic criteria or either definite or “atypical” MD, and from these, there were 78 control ears derived according to the previously stated conditions.

### Inter-rater reliability

The inter-rater reliability for the MRI descriptors (*n* = 454) is documented in Table 4. “Saccule as large as or confluent with the utricle” demonstrated the highest kappa value of *κ* = 0.920 (0.881–0.959) with very good agreement. The poorest agreements were recorded for grade 1 cochlea with *κ* = 0.511 (95% confidence interval (CI), 0.429–0.593) and “33–50% VES relative to total vestibular (TV) area (superior)” with *κ* = 0.505 (95% CI, 0.417–0.593). There was a positive correlation between the two observers for quantitative cochlear PLE with *r*_*s*_ = 0.858 (*p* < 0.001). All VA descriptors demonstrated very good reliability of *κ* = 0.801–0.868. The weighted kappa (*κw*) values were 0.727 (0.672–0.783) for the Nakashima scale, 0.844 (0.787–0.900) for the Barath scale, 0.840 (0.796–0.884) for the Bernaerts scale and 0.818 (0.775–0.862) for the Kahn scale.

**Table 4:** Inter-rater reliability kappa values, sensitivity/specificity and DOR for the MRI descriptors

	Cohen’s kappa (95% CI)	Sensitivity/specificity (%)	Diagnostic odds ratio (DOR) (95% CI)
Cochlear			
Grade 1	***κ* = 0.511 (0.429–0.593)**	82.3/87.2	31.6 (13.564–73.617)
Grade 2	*κ* = 0.852 (0.791–0.913)	69.8/96.2	**57.759 (16.824–198.297)**
Asymmetric cochlear PLE	*κ* = 0.769 (0.697–0.842)	62.5/94.9	30.833 (10.391–91.493)
Superior vestibule			
> 33% VES area relative to TV area (superior)	***κ* = 0.505 (0.417–0.593)**	72.9/82.1	12.308 (5.914–25.612)
> 50% VES area relative to TV area (superior)	*κ* = 0.846 (0.777–0.916)	51.0/98.7	**80.277 (10.726–600.827)**
No lateral SCC ampullary PS visible	*κ* = 0.761 (0.618–0.904)	20.8/98.7	20.263 (2.653–154.782)
Lateral SCC posterior limb ES extension	*κ* = 0.740 (0.646–0.838)	43.8/96.2	19.444 (5.727–66.021)
PS not visible (superior)	*κ* = 0.649 (0.484–0.814)	17.7/100	1.215 (1.108–1.333)
Inferior vestibule			
Saccule absent, as large as or confluent with the utricle	*κ* = 0.920 (0.881–0.959)	79.2/98.7	**292.6 (38.305–2235.058)**
Saccule as large as or confluent with the utricle	*κ* = 0.938 (0.900–0.975)	71.9/98.7	**196.778 (26.046–1486.644)**
Saccule confluent with the utricle	*κ* = 0.898 (0.845–0.951)	68.8/98.7	**169.4 (22.487–1276.107)**
> 50% VES area relative to TV area (inferior)	*κ* = 0.924 (0.877–0.971)	64.6/98.7	**140.412 (18.692–1054.776)**
PS not visible (inferior)	*κ* = 0.685 (0.540–0.830)	20.8/98.7	20.262 (2.653–154.782)
VES contacting the oval window	*κ* = 0.833 (0.766–0.833)	61.5/98.7	**122.784 (16.369–920.980)**
Vestibular aqueduct			
Non-visualised VA	*κ* = 0.812 (0.751–0.873)	89.6/47.4	7.761 (3.517–17.125)
Incompletely visualised VA	*κ* = 0.868 (0.821–0.913)	85.4/75.6	18.1 (8.445–39.170)
No hyperintensity VA	*κ* = 0.801 (0.741–0.861)	59.4/88.5	11.205 (5.009–25.068)

*VES* vestibular endolymphatic space, *TV* total vestibular, *PS* perilymphatic space, *SCC* semi-circular canal, *PLE* perilymphatic enhancement, *VA* vestibular aqueduct, *ES* endolymphatic space

Kappa values < 0.6 and DOR > 50 in bold

### Diagnostic performance of individual descriptors and parameters

All MRI descriptors were associated with the presence of definite MD ears (*p* < 0.001). The sensitivity, specificity and DORs of the MRI descriptors for the detection of definite MD ears are shown in Table 4. The highest DOR of 292.6 was achieved by “saccule absent, as large as or confluent with the utricle” (Fig. 2).

The DOR for the novel VA descriptors ranged from 7.761 to 18.1 and was highest for incompletely visualised VA (Figs. 3 and 4). There were 45/96 MD (16 CT, 19 T2w MRI, 10 both) and 58/78 control (10 CT, 34 T2w MRI, 14 both) ears in which either CT or T2w MRI clearly demonstrated VA for anatomical verification.

The quantitative cochlear PLE measurements were significantly increased in definite MD ears compared to control ears according to the Mann-Whitney *U* test (*U* = 6072; *z* = 7.04; *p* < 0.001) with AUC = 0.811 (0.748–0.873) indicating excellent discrimination.

The Nakashima (*χ*^2^ = 60.796, *p* < 0.001), Barath (*χ*^2^ = 72.762, *p* < 0.001), Bernaerts (*χ*^2^ = 87.029, *p* < 0.001) and Kahn score (*χ*^2^ = 86.059, *p* < 0.001) grades were all significantly associated with the presence of definite MD ears.

### Logistic regression

Of the initial 17 MRI descriptors and one quantitative measure, two were excluded from the analysis due to kappa values < 0.6 (grade 1 cochlea and “33–50% VES relative to TV area (superior)”) and three due to collinearity (“> 50% VES area relative to TV area (inferior)”, “saccule as large as or confluent with the utricle”, “saccule confluent with the utricle”). Four standardised residuals with values of greater than 2.5 standard deviations (SDs) were kept in the analysis.

**Fig. 6:**
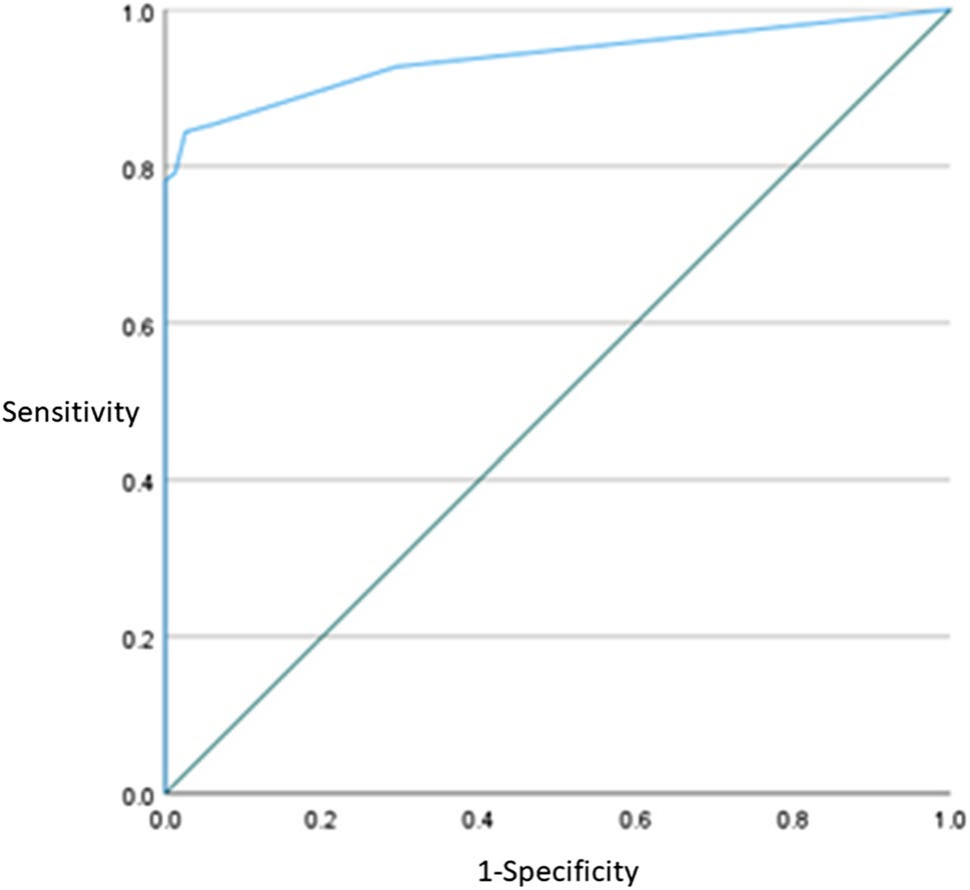
Receiver operating characteristic (ROC curve) for the model comprising “saccule absent, as large as or confluent with the utricle”, “asymmetric cochlear PLE” and “incompletely visualised vestibular aqueduct”

Of the remaining 13 MRI variables, only three were statistically significant (Table 5): “saccule absent, as large as or confluent with the utricle” (*χ*^2^ = 129.957, *p* < 0.001), “asymmetric cochlear PLE” (*χ*^2^ = 10.422, *p* < 0.001) and “incompletely visualised VA” (*χ*^2^ = 8.280, *p* < 0.001). The model explained 76.9% (Nagelkerke *R*^2^) of the variance in definite MD and correctly classified 90.2% of cases (sensitivity 84.4%, specificity 97.4%, PPV 97.6%, NPV 83.5%). The AUC was 0.938 (95% CI, 0.900–0.976) (Fig. 6) which is an outstanding level of discrimination [33]. Repeating the logistic regression after removing MD ears which had undergone ITT yielded the same three significant MRI descriptors, correctly classifying 90.9% of cases (sensitivity 85.1%, specificity 97.4%, PPV 97.4%, NPV 85.4%).

**Table 5:** Variables in the equation showing the contribution of each of the three MRI descriptors to the model and its statistical significance

	*B* coefficient	SE	Wald	*p*-value	Odds ratio	95% CI for odds ratio	
						Lower	Upper
Saccule absent, as large as or confluent with the utricle	4.410	1.066	17.111	< 0.001	82.300	10.182	665.214
Asymmetric cochlear PLE	1.943	0.726	7.160	.007	6.979	1.682	28.962
Incompletely visualised VA	1.561	0.546	8.168	.004	4.762	1.633	13.885

*PLE* perilymphatic enhancement, *VA* vestibular aqueduct, *SE* standard error

## Discussion

All MRI descriptors were recorded with good to excellent reliability apart from grade 1 cochlear and “33–50% vestibular endolymphatic space relative to total vestibular area (superior)”. Both are derived from the Nakashima grading system (*κw* = 0.727) which was less reliable than those of Barath, Bernaerts and Kahn (*κw* = 0.818–0.844). The DORs for the MRI descriptors ranged from 1.215 to 292.6. Those derived from the inferior vestibule performed best, and “saccule absent, as large as or confluent with the utricle” had the largest DOR of 292.6 (95% CI, 38.305–2235.058), although there were wide confidence intervals. The newly defined VA descriptors all had excellent reliability and demonstrated DORs of 7.761 (95% CI, 3.517–17.125) to 18.1 (95% CI, 8.445–39.170). A forward stepwise logistic regression demonstrated the presence of “saccule absent, as large as or confluent with the utricle” or “asymmetric cochlear PLE” and “incompletely visualised VA” to be the statistically significant variables, correctly classifying 90.2% of cases (sensitivity 84.4%, specificity 97.4%, PPV 97.6%, NPV 83.5%).

There has been no previous integrated comparison of reliability and performance applied to a comprehensive range of delayed post-gadolinium MRI descriptors for the diagnosis of MD. A recent systematic review found only 3/72 studies [1, 2, 7, 8] applying more than one vestibular grading scale. These three studies evaluated both SURI (inversion of the saccule to utricle area ratio) and the Nakashima grading scale, but sample sizes were small (*n* ≤ 30). Two further recent studies have compared the reliability and diagnostic performance of different vestibular grading systems, but not individual descriptors. The Bernaerts scale was found to be more sensitive than Barath [9], whilst Xiao et al found the authors’ own 18-point scoring scale to have superior diagnostic performance to the Bernaerts grade [10].

The consideration of newly defined VA descriptors was motivated by previous histopathological [13, 14] and radiological studies [16–20] which have highlighted associations between a reduction in size and conspicuity of the VA and endolymphatic sac in MD ears. Attyé et al evaluated the visibility of the VA in a cohort of 20 unilateral MD ears compared to 20 healthy control patients and found a bilateral reduction in visibility in unilateral MD [19]. Our study applied similar imaging analysis and confirmed that “incompletely visualised” was reliably evaluated and added value to established MRI descriptors by its contribution to the logistic regression model. In addition, we demonstrated the absence of hyperintensity within the VA to be a predictor of MD ears with an odds ratio of 11.205 (5.009–25.068). Hyperintensity due to VA enhancement has been observed on early post-gadolinium MRI, in both normal ears [21] and in immune-mediated and viral inner ear diseases [22, 23]. It is speculated that our observation of absent VA hyperintensity is due to reduced gadolinium enhancement, and this may be related to the fibrotic changes which develop within the richly vascularised [34] peri-saccular loose connective tissue [14] in MD.

Our study outcomes build on previous work [4, 11] identifying SURI or higher grade EH and PLE as key MRI predictors for definite MD. These previous studies applied the asymptomatic contralateral ear in unilateral MD as the control, which is not ideal since EH has been observed to be present in up to 23.3% of asymptomatic contralateral ears in MRI studies [35] as well as in histopathology studies [36]. The control ears in our study were all from asymptomatic ears in patients without any audiometric or vestibular features suggestive of MD. The potential for our exploratory descriptor “absent saccule” as a marker of MD was first highlighted by Attyé [2] but it has not been evaluated in a large cohort for its diagnostic potential. Whilst an absent saccule due to rupture or fistula is infrequently associated with definite MD ears [37], our study has demonstrated it to provide improved diagnostic performance when added to the other saccular descriptors.

This study has limitations that should be considered. Firstly, there is innate bias in patient selection introduced by the case-controlled study design [38]. Although inappropriate exclusions such as bilateral MD and ITT interventions were avoided, nine ears were excluded post hoc due to technically inadequate imaging, which may distort outcomes. Secondly, the retrospective nature of the study led to an interval between the imaging index test and the clinical MD reference standard. These concerns may be addressed by future application of a prospective study design. Thirdly, although the MRIs were interpreted blinded to the clinical diagnosis, there was potential bias due to the unavoidable viewing of multiple MRI descriptors simultaneously within each ear. Fourthly, the generalisability of the results may be restricted since the performance of the MRI descriptors may depend on the specifics of the MRI sequence and technique. There is also no guarantee that the selection of optimal MRI descriptors would be the same in patients with atypical phenotypes of MD. Subgroup analysis was limited by sample size in the current study. In addition, a number of the estimates of diagnostic accuracy demonstrated wide confidence intervals, which should hence be interpreted with caution. Fifthly, the range of MR descriptors studied was not exhaustive. It was decided a priori to study a single cochlear hydrops grading system since there are no substantial differences between the different grading scales, and descriptors employed in less than three studies were excluded. Finally, with respect to the novel VA descriptors, anatomical correlation was not always possible due to CT not being available or poor visualisation on T2w MRI. Considering it was hypothesised that a reduction in conspicuity was associated with MD ears, the exclusion of these ears from the analysis may have led to partial verification bias.

A direct comparison of MRI descriptors for the diagnosis of definite MD ears found 15/17 to be demonstrated with good to excellent reliability and with “saccule absent, as large as or confluent with the utricle” demonstrating the best diagnostic performance. The three VA MRI descriptors were reliable and demonstrated high DORs. “Incompletely visualised VA” was included in the optimal combination of three descriptors for the diagnosis of MD ears, indicating its added value. The radiologist should focus on these three highly performing descriptors in combination when reporting delayed post-gadolinium MRI, to best corroborate the diagnosis of MD. As guidelines develop which incorporate MRI in their diagnostic criteria for MD [39], specific imaging criteria may now be advised.

### Supplementary Information

Below is the link to the electronic supplementary material.

Supplementary file1 (PDF 297 KB)

## Abbreviations

AUC: Area under the curve
CI: Confidence interval
CT: Computed tomography
DOR: Diagnostic odds ratio
EH: Endolymphatic hydrops
ES: Endolymphatic space
IR: Inversion recovery
ITT: Intratympanic therapy
MD: Ménière’s disease
MRI: Magnetic resonance imaging
PLE: Perilymphatic enhancement
PS: Perilymphatic space
SCC: Semi-circular canal
SPACE: Sampling perfection with application of optimised contrasts using different flip angle evolutions
SURI: Saccule to utricle area ratio inversion
TV: Total vestibular
VA: Vestibular aqueduct
VES: Vestibular endolymphatic space

## Declarations

### Guarantor

The scientific guarantor of this publication is Dr Steve Connor.

### Statistics and biometry

One of the authors has significant statistical expertise.

### Study subjects or cohorts overlap

The patient cohort in this study has an overlap with that applied to three previous studies. The hypothesis and analysis are however different. There are a total of 44 individual patients of the 227 patients in the current cohort who were included in the overlapping cohorts of three previous studies:

31 patients in

Pai I, Mendis S, Murdin L, Touska P, Connor S. Magnetic resonance imaging of Ménière’s disease: early clinical experience in a UK centre. J Laryngol Otol. 2020 Apr;134(4):302-310. 10.1017/S0022215120000626. Epub 2020 Apr 3. PMID: 32241307.

22 patients in

Connor S, Hulley K, Burd C, Amin N, Pai I. Which is the optimally defined vestibular cross-section to diagnose unilateral Meniere’s disease with delayed post-gadolinium 3D fluid-attenuated inversion recovery MRI? Br J Radiol. 2021 Mar 1;94(1119):20200996. 10.1259/bjr.20200996. Epub 2021 Jan 15. PMID: 33449817; PMCID: PMC8011242.

35 patients in

Pai I, Connor S. Low frequency air-bone gap in Meniere’s disease: relationship with magnetic resonance imaging features of endolymphatic hydrops. Ear Hear. 2022 Nov-Dec 01;43(6):1678-1686. 10.1097/AUD.0000000000001231. Epub 2022 May 18. PMID: 35583512; PMCID: PMC9592161.

### Methodology

• retrospective

• case-control study

• performed at one institution
