# Supplementary material for: Assessing the optimal MRI descriptors to diagnose Ménière’s disease and the added value of analysing the vestibular aqueduct
Source: Eur Radiol. 2024 Feb 7;34(9):6060–71. doi: 10.1007/s00330-024-10587-w (PMC11364795; doi:10.1007/s00330-024-10587-w)
Supplement: Supplementary file 1 — Supplementary file1 (PDF 297 KB) [file 330_2024_10587_MOESM1_ESM.pdf]

Assessing the optimal MRI descriptors to diagnose Ménière's disease and the added value of analysing the vestibular aqueduct

Electronic Supplementary Material (ESM)

Eur Radiol (2024) Connor S, Pai I, Touska P, McElroy S, Ourselin S, Hajnal JV

Supplementary Table 1: Clinical criteria used to define Ménière’s disease

Clinical classification	Clinical criteria
Definite 2015* Barany Society clinical *Criteria for MD (24)	
	A. ≥2 spontaneous episodes of vertigo, each lasting 20 minutes to 12 hours<br>B. Audiometrically documented low- to medium-frequency SNHL in affected ear on ≥1 occasion before, during or after 1 of the episodes of vertigo<br>C. Fluctuating aural symptoms (hearing, tinnitus or fullness) in affected ear<br>D. Not better accounted for by another vestibular diagnosis
Other clinical criteria for MD or hydropic ear disease (“Atypical” MD)	
**2015 Ménière’s disease Criteria (24)**	
Probable	A. ≥2 episodes of vertigo or dizziness, each lasting 20 minutes to 24 hours<br>B. Fluctuating aural symptoms (hearing, tinnitus or fullness) in affected ear<br>C. Not better accounted for by another vestibular diagnosis
**1995 Ménière’s disease Criteria (27)**	
Certain Ménière’s disease	Definite MD, plus histopathologic confirmation
Definite Ménière’s disease	A. Two or more definitive spontaneous episodes of vertigo 20 minutes or longer<br>B. Audiometrically documented hearing loss on at least one occasion<br>C. Tinnitus or aural fullness in the treated ear

	D. Other causes excluded
Probable Ménière's disease	A. One definitive episode of vertigo<br>B. Audiometrically documented hearing loss on at least one occasion<br>C. Tinnitus or aural fullness in the treated ear<br>D. Other causes excluded
Possible Ménière's disease	A. Episodic vertigo of the Ménière type without documented hearing loss, or<br>B. Sensorineural hearing loss, fluctuating or fixed, with dysequilibrium but without definitive episodes<br>C. Other causes excluded
**Monosymptomatic cochlear hydrops (cMD)**	
1972 Criteria (25)	Characterized solely by a fluctuating and progressive sensorineural deafness with all auditory test results typical of MD. Many patients notice a fullness in the ear coincident with the sudden drop in hearing. Some subsequently develop definitive dizzy spells, and the qualifying ''cochlear'' is discarded
Kimura et al (30)	Fluctuating hearing loss with single episode of vertigo, unsteadiness, or no vestibular symptoms
2020 Japanese Clinical Practice Guideline of Ménière's Disease (29)	Recurrent cochlear symptoms (eg hearing loss, tinnitus, aural fullness) without vertigo attacks with audiometrically demonstrated SNHL (usually low frequency or pan-frequency)
Gurkov (28)	Acute onset low tone acute low tone sudden onset SNHL
Summary criteria for cochlear hydrops applied	Fluctuating hearing loss or acute low tone sudden onset SNHL with or without aural fullness/tinnitus but no MD type vertigo (eg only single attack of vertigo alone, or with concurrent unsteadiness).
**Monosymptomatic vestibular hydrops (vMD)**	

1972 Criteria (25)	Characterized solely by definitive spells of vertigo. This is more difficult to diagnose as there are no objective findings between spells. The diagnosis may be accepted upon exclusion of other diseases. Some patients subsequently develop deafness, and the qualifying ''vestibular'' is dropped
Kimura et al (30)	Recurrent episodic vertigo with or without fixed hearing loss
2020 Japanese Clinical Practice Guideline of Ménière's Disease (29)	Recurrent attacks of vertigo typical for MD and signs of peripheral dysfunction without accompanying fluctuating cochlear symptoms
Summary criteria for vestibular hydrops applied	MD type vertigo/episodic vertigo with or without aural fullness/tinnitus and with or without fixed SNHL (but not fluctuating or low frequency).
Control ears	
	Neither ear satisfying clinical criteria for MD on any previous classification or any features of hydropic ear disease (as above)
	Audiogram: Normal thresholds (between 0-25dB at all frequencies) or isolated high frequency hearing loss (>6K)
	No MD type vertigo (recurrent episodic and lasting 20 minutes to 24 hours)
	Aural fullness or tinnitus permitted if it could be explained by an alternative diagnosis (e.g eustachian tube dysfunction)

MD = Ménière's disease; SNHL = sensorineural hearing loss

Supplementary Table 2: Description of MRI grading scales incorporating the MRI descriptors

	Nakashima (6)	Barath (3)	Bernaerts (4)	Kahn (5)	Increased peri-lymphatic enhancement (PLE) (7)
**Cochlea**					
Plane and location	**Axial**: Mid-modiolar level	**Axial:** Mid-modiolar level	**Axial:** Mid-modiolar level	**Axial:** Mid-modiolar level	**Axial:** Basal turn and typically inferior segment
Imaging feature/grading	*Grade 1*: cochlear duct area < scala vestibuli area<br>*Grade 2*: cochlear duct > scala vestibuli area*	*Grade 1*: cochlear duct spares part of scala vestibuli<br>*Grade 2:* cochlear duct replaces scala vestibuli*	*Grade 1:* Nodular cochlear duct enlargement "Xmas tree balls"<br>*Grade 2:*<br>Linear cochlear duct enlargement "Xmas garlands" *	Cochlear duct > scala vestibuli area	Asymmetrically increased (in unilateral Meniere's disease) or subjectively increased cochlear PLE
**Vestibule**					

Plane and location	**Axial:**<br>Inferior aspect of lateral semi-circular canal	**Axial:**<br>At widest part of the vestibule (inferior)	**Axial**:<br>At widest part of the vestibule (inferior)	**Axial**:<br>Variable (superior and inferior vestibule)	NA
Imaging feature/grading	*Grade 1:* % area of endolymph relative to total fluid area 33-50 %<br>*Grade 2:* % area of endolymph relative to total fluid area >50%	*Grade 1:* >50% area of endolymph relative to total fluid area<br>*Grade 2:* enhancing perilymphatic space of the vestibule not visible	*Grade 1:* SURI<br>*Grade 2:* utricle and saccule are confluent<br>Grade 3: enhancing perilymphatic space of the vestibule not visible	**Saccule:**<br>*Grade 1:* SURI<br>Grade 2: touching oval window<br>**Utricle:**<br>*Grade 1:* herniation to lateral SCC<br>*Grade 2:* enhancing perilymphatic space of the vestibule not visible<br>**Ampulla:** No surrounding perilymphatic space	NA
